# How Race Matters for Elites' Views on Redistribution

**DOI:** 10.1111/1468-4446.70012

**Published:** 2025-08-12

**Authors:** Chana Teeger, Livio Silva‐Muller, Graziella Moraes Silva

**Affiliations:** ^1^ Department of Methodology London School of Economics and Political Science London UK; ^2^ Department of Sociology University of Johannesburg Johannesburg South Africa; ^3^ Albert Hirschman Centre on Democracy and Department of Anthropology & Sociology Geneva Graduate Institute Geneva Switzerland

**Keywords:** collective memory, elites, race, redistribution, South Africa

## Abstract

Elites are increasingly visible in academic and political discourse owing to their disproportionate power in shaping policy. For the most part, however, elites have been viewed in race‐blind terms. In this paper, we advance a racialized perspective on elite studies by highlighting three salient ways that race matters for elite views on inequality and redistribution. First, we focus on elites as racialized actors whose racial identities impact their perspectives on social policies. Second, we examine the effect of holding a historical perspective of racialized inequality on elites' redistributive preferences. Third, we highlight the importance of attending to the racialization of social policies, distinguishing between redistributive measures framed in race‐neutral and race‐conscious terms. We demonstrate the utility of a racialized approach to elite studies by analyzing survey data collected from political, economic, and civil service elites in South Africa. Findings show that elites' racialized identities shape their redistributive preferences, as do their historical understandings of racialized inequality, but these effects vary depending on whether elites are evaluating race‐conscious or race‐neutral policies.

## 1 Introduction

Rising inequality has brought with it renewed attention to elites' disproportionate power in shaping policy (López et al. 2022; Reeves and Friedman 2024; Reis and Moore 2005). Testing common‐sense notions that elites will always oppose redistribution, researchers have sought to identify elites' redistributive preferences by focusing on specific state‐led policies aimed at reducing inequality.^1^ They have shown that elites' views on redistribution are not monolithic and that elites are indeed willing to support a range of redistributive policies under certain conditions (López 2023b; de Swaan 1988).

For the most part, however, the literature on elites' views of inequality and redistribution has been relatively race‐blind. When researchers have attended to race, they have tended to focus narrowly on elites' own racial identifications, often using these data as control variables in statistical models. Race has not featured predominantly as an analytical category through which to understand elite perceptions and policy orientations.

In what follows, we argue for a broader conceptualization of the role of race in elites' redistribution preferences. While elites' own racial identification is highly relevant, racialization processes go beyond individual identity. We argue that researchers can attend to these broader processes in two main ways: first, by looking at elites' historical understandings of racialized inequality; and second, by attending to the racialization of policies. This perspective moves us away from static and essentialized notions of race and toward a recognition of racialization processes. By attending to when and how elites' racialized identities matter in relation to other processes of racialization (i.e., *historical understandings of racialized inequality* and the *racialization of policies*), we can begin to unpack the complex ways that race shapes elites' redistributive preferences.

We develop this argument by focusing on South Africa. South Africa is a particularly generative case for the study of elites, race, and redistribution preferences. The comparative race relations literature has historically taken South Africa to represent a case of extreme and rigid racial boundaries and hierarchies (Fredrickson 1988; Hamilton 2001; Lieberman 2003; Marx 1998). However, since the dismantling of apartheid, such boundaries have begun to blur, with the growth of a Black middle class and elite (Southall 2016) and the massive increase in intra‐racial inequality (Seekings and Nattrass 2008), as well as the implementation of a set of race‐conscious policies known as Black Economic Empowerment (BEE) (Tangri and Southall 2008). These trends have been accompanied by powerful discourses of color‐blindness (Ansell 2004; Steyn 2001; Teeger 2015)—though these are not without challenge (see Teeger 2024).

Our paper asks how an increasingly racially diverse elite thinks about redistribution, and whether and how race matters in these considerations. Empirically, we draw on an original survey fielded among business, civil service, and political elites in South Africa in 2014 (*N* = 184) and 2023 (*N* = 155), where we asked respondents a range of questions aimed at tapping their views on inequality and redistribution.

Following a strategy that draws on Hoffmann‐Lange's (2018) positional method, we randomly sampled elites based on their institutional positions. We focused on business leaders, elected officials at the national level, and top‐tier civil servants. We chose these elites because of their decision‐making power and ability to shape redistributive policies. Elected officials have an obvious influence on policy once it is in their hands to put forward legislation. Civil servants hold significant power over policymaking (Evans 1995). Finally, the influence of economic elites on policymaking and agenda‐setting processes is well documented (García‐Montoya and Manzi 2023; Gilens and Page 2014).

In analyzing our data, we center race in three distinct ways. First, we focus on respondents' own racialized identities. Second, we look at respondents' understandings of racialized inequalities. Here, we examine whether respondents view current racialized inequalities as reflecting histories of racial oppression and privilege. Third, we attend to the racialization of social policies, distinguishing between policies framed in race‐neutral terms and those that are explicitly race‐conscious. We examine the effects of (a) elites' racialized identities and (b) their historical understanding of racialized inequality on their support for race‐neutral and race‐conscious policies.

Our findings show that Black African elites are more supportive of race‐conscious redistributive policies than are White elites. At the same time, respondents across racial groups who believed in the continued effects of apartheid on contemporary South Africa were more likely to support race‐conscious policies than were those who did not adopt this historical frame. We outline the implications of these findings for literature on elites, racism, and collective memory.

## 2 Elites’ Views on (In)Equality

Studies of elites' views on inequality trace back to Verba and colleagues' work, which pioneered surveys of elites—first in the United States (Verba and Orren 1985) and then comparatively in Japan, Sweden, and the United States (Verba et al. 1987). These surveys focused on two dimensions of equality. First, they asked about elite perceptions of economic equality; that is, what elites view as fair or unfair drivers of income differences. Second, they examined elites' perceptions of political equality. Here, they analyzed elites' views on citizens' access to—and influence over—the state beyond their economic power. In their comparative work, Verba et al. (1987) explained differences between countries as resulting from divergent political cultures and the historical meaning of equality. Variations within countries were explained by elite sector.

Reis and Moore (2005) took inspiration from this work, bringing the empirical focus to the global South and moving the analysis from *equality* to poverty and *inequality*. Highlighting cross‐national differences in support for redistribution, Reis and Moore (2005:8) echo Verba et al. by arguing that “the stuff of which culture is made—“habits of the heart”—is extremely resilient.” However, following de Swaan (1988), they also argue that elites will support redistribution under the following conditions: (1) If they believe that poverty has negative effects not only for the poor, but also for themselves; (2) If they believe that they have some responsibility toward the poor; and (3) If they believe that there are mechanisms to reduce poverty.

Several studies have followed that attempt to identify the determinants of elites' support for redistribution, broadly understood. Researchers have demonstrated a robust association between elites' fear of crime and their redistributive preferences (Rueda and Stegmueller 2016). They have also shown how elites' views of merit and deservingness impact their willingness to redistribute (Atria et al. 2020; Kuusela 2022; Sherman 2020), as does their view of the poor as political actors (López et al. 2022). Beyond the effect of party affiliation (Bartels 2016; Page et al. 2013), researchers have shown how trust in institutions (Weinberg 2023) and views on state efficiency (Fisman et al. 2015) matter for elites' support for redistribution.

This literature has been relatively silent on questions of race. While race is sometimes used as a control variable, researchers have yet to fully consider how race shapes elites' views on redistribution (see Cousin et al. 2018; J. Hochschild 2009). This is hardly surprising. In racially stratified societies, there tends to be little racial variation at the top (see Young et al. 2021). Still, elites are not homogenous. While Whites continue to dominate in positions of power in the global economy, processes of decolonization and democratization have brought non‐White elites into power in Africa, Asia, and Latin America (Mahler 2018; Schneickert et al. 2015). In the global North, too, the elite is becoming increasingly racially diverse (see, e.g. Reeves and Friedman 2024; Zweigenhaft and William Domhoff 2006). Moreover, as discussed next, researchers have pointed to the centrality of race in our understanding of elite formations in different historical times and places.

## 3 Race and Elites

While research on elites' perceptions of inequality and redistribution has not focused much on race, the broader literature has homed in on the centrality of race for elite formation and reproduction. State‐building scholarship has shown how competition between elite groups led to distinctive racial formations in different countries (e.g., Johnstone 1976; Marx 1998). Like the literature on elites' perceptions of inequality, discussed above, this state‐building literature highlights differences and divisions within national elites. But the latter goes one step further, showing how White elites consolidate their power—despite regional and ethnic differences—through the formation of a racial regime that puts them at the top.

Others have focused on elite spaces as racialized organizations (Ray 2019), centering the experiences of racialized minorities within them. These studies reveal the white logics that underpin these spaces (Ispa‐Landa and Conwell 2015) and the discrimination and misrecognition that racialized minorities experience within them (Lamont et al. 2016; Moraes de Silva and Reis 2011). Indeed, elite status can heighten experiences and awareness of discrimination as racialized minorities learn that wealth and power do not obliterate racism (J. L. Hochschild 1996; Pittman Claytor 2020). Such experiences can lead to perceptions of “linked fate” between racialized minorities in different socio‐economic positions, generating racial identification and solidarity that transcends class lines (Lacy 2007; Lu and Jones 2019).

At the same time, increased racial and ethnic diversity among the elite does not necessarily reflect, or lead to, more racially progressive politics—as the rise of racial and ethnic minority politicians in historically White conservative groups attests. Such individuals have promoted ideas of meritocracy and individual merit, denying the effects of historical or contemporary discrimination and often opposing race‐conscious policies (Fields 2016; Saini et al. 2023). How elites' racial identities and mobility trajectories structure their political views is an empirical question that might have different answers in different times and places (see Moraes Silva 2020).

Acknowledging the potential for variation in how elites' own racialized identities structure their redistributive policy preferences means moving beyond racial identity as a static category by considering how individuals *interpret racial inequalities* in their societies. While individuals' racialized identities are often correlated with their understanding of the salience, causes, and responses to racial inequalities (see Farrington 2016; Teeger 2014), this is not always the case (see Bonilla‐Silva 2006; Teeger 2024).

We draw on these insights to examine three analytically distinct ways in which race matters for elites' redistribution preferences. First, following the literature mentioned above, we expect elites' *racialized identities* to affect their support for redistribution. Second, we expect elites' *historical understandings of racialized inequality*—this is, their views on the extent to which histories of racism are a salient factor in explaining contemporary inequality—to impact upon their support for redistributive policies. Third, we expect this support to vary based on the *racialization of policies*; that is, whether the policies are framed in race‐conscious or race‐neutral terms. Specifically, drawing on the “linked fate” literature (Lacy 2007; Lu and Jones 2019), we hypothesize that elites will support policies targeted at individuals who share their racialized identities. Drawing on the collective memory literature (Teeger 2014, 2024), we hypothesize that elites who hold a historical view of racialized inequality will be more likely to support race‐conscious policies than elites who do not hold this historical understanding.

## 4 Data and Methods

### 4.1 Case Selection

We view South Africa as a particularly generative case through which to think about elites, race, and redistribution for two main reasons. First, since the end of the apartheid in the 1990s, Black elites have occupied most positions of power at the political level, while economic power is still largely concentrated in the hands of White elites. This means that South Africa is one of the few countries with substantial racial variation at the top, even if the racial distribution is uneven across sectors and the diversity among the elite is not mirrored at the other end of the distribution, where the poor are almost entirely Black (Seekings and Nattrass 2008, 2015).^2^

Second, since the end of apartheid, the South African government has implemented a range of redistributive measures. Some of these, like social grants (a cash transfer program), are framed in race‐neutral terms. Others, like BEE, are framed in explicitly race‐conscious terms. At the same time, redistributive policies are often debated in the public sphere; e.g., during the 2015 FeesMustFall protests, when students protested racially exclusionary university fees by calling for their universal (i.e. race‐neutral) abolition (see Godsell et al. 2016) or with the emergence of new political parties (such as the Economic Freedom Fighters) who call for race‐conscious land redistribution and wealth transfer policies (see Kepe and Hall 2020). Debates about these policies often draw links between the country's apartheid past and contemporary structures of inequality. As such, South Africa represents a case where elites have actively sought to implement both race‐neutral and race‐conscious redistributive policies, but they have done so within the context of persistently high levels of inequality and under the shadow of a history of legislated racism that continues to structure contemporary inequality. Our study asks how race shapes elites' own views on redistribution.

### 4.2 Survey

To address this question, we draw on data we collected for a broader study on elites' perceptions of inequality and redistribution in the global South. We fielded an elite survey in South Africa in 2014 (*N* = 184) and 2023 (*N* = 155). Following a strategy that draws on Hoffmann‐Lange's (2018) positional method, we randomly sampled elites based on their institutional positions. The positional definition of elites varies from income/wealth or reputational definitions, which are more common in studies of elite reproduction.^3^ Since our interest is in redistribitive social policies, we focused on individuals who occupy powerful institutional or organizational positions in relation to the implementation of such policies. We randomly sampled elites by sector focusing on: (1) political party representatives; (2) top‐tier civil servants; and (3) business leaders. This of course is not an exhaustive list of elite sectors. For example, cultural elites, such as high‐ranking figures in the arts or academia, play an important role in shaping public discourse. However, their ability to effect redistributive policies is more subtle than that of respondents in our sample, who represent elite factions associated with economic and political power.

Our sample of political elites includes elected officials from the two largest parties in Parliament (the National Assembly): the African National Congress (ANC) and the Democratic Alliance (DA).^4^ For our sample of civil servants, we strategically chose 20 government departments charged with a range of fiscal and social policy mandates and selected randomly from top‐tier roles (Director Generals, Deputy Director Generals, Chief Operating Officers, and Chief Directors). To construct our sample of business leaders, we triangulated a list of the top 300 private firms (by market capitalization) from the Johannesburg Stock Exchange with The Africa Report's list of top 500 companies, which had been identified as a widely used list by a senior contact at the Competitions Commission. We randomly sampled companies from this list, allowing individual respondents to be drawn from one of the following positions: CEO, CFO, or Chairperson of the Board.

Our 2014 survey tapped a range of elites' views on poverty, inequality, democracy, and a variety of groups and institutions. Informed by findings from our original survey, our 2023 survey reproduced questions from our 2014 survey but also introduced several new ones aimed at testing hypotheses about the determinants of elites' policy preferences. Among the questions we asked in both surveys was a battery of questions about the effects of apartheid on contemporary society, which we utilize in this paper.

Surveys were administered by fieldworkers in person in 2014 and remotely in 2023. We opted against self‐administered surveys to ensure that completion of the survey would not be delegated to aides. In so doing, we follow López's (2023a) assessment that probability sampling with face‐to‐face survey administration is a particularly high standard for elite surveys. Still, the fieldworker‐administered survey format introduces concerns around social desirability bias, in particular when measuring elites' views on redistributive policies. We see this as a trade‐off. We preferred to ensure that we captured elites' own views—even if these are affected by the relational dimension of the data collection method—rather than risk attributing to elites views that were not their own. As discussed below, we combined a measure of elites' assessment of the desirability and viability of social policies to mitigate against social desirability biases.

As noted, we sampled participants positionally, interviewing whomever held a particular position in each of the two time periods. We did not set out to reinterview respondents from 2014 in 2023. Table 1 summarizes response rates and sample sizes per sector for both waves.

**TABLE 1:** Sample size and response rate by sector and year.

Year	Business	Political	Civil servants	Total
2014	61 (32%)	63 (65%)	60 (35%)	184 (44%)
2023	51 (24%)	71 (32%)	33 (17%)	155 (25%)

Several factors could account for the different response rates between the two waves. The 2023 survey was administered online in the aftermath of the Covid‐19 pandemic. Increased remote working patterns and the associated “Zoom fatigue” may have reduced elites' willingness to log in for an interview. Somewhat related, in 2014, the survey company we hired sent fieldworkers to Cape Town during parliamentary sittings to recruit MPs for our elected officials sample. We believe that this recruitment strategy helped generate the high response rate for this sub‐sample in 2014. The civil service sample was the most difficult to recruit in both waves, as it was difficult to find information about the particular people occupying specific positions. In 2014, fieldworkers often went in person to try to identify the relevant individuals, which helped boost the size of this sub‐sample. Finally, in the 2014 sample, we aimed for approximately 60 participants in each sub‐sample. We provided the survey company with the list of sampled individuals in stages, waiting for them to exhaust initial contacts before providing more names. In 2023, we provided the full list of sampled individuals up front and did not give a target for each sub‐sample. Instead, like in 2014, we insisted that participants' could only be categorized as non‐responders after three failed contact attempts. The number of names provided in both periods was similar, and—given that coefficients are largely consistent in magnitude and direction across the two years—we do not have reason to believe that our findings are sensitive to the minor differences in survey protocols between waves. We account for the possibility of such differences by controlling for survey year in our statistical models.

### 4.3 Operationalization and Data Analysis

#### 4.3.1 Outcome Variables

We have four outcome variables, each representing respondents' level of support for a distinct redistributive policy. The policies include an explicitly race‐conscious policy and three race‐neutral policies that vary in their target population and level of universality. The policies are as follows:

- *Black Economic Empowerment (BEE).* BEE is an explicitly race‐conscious policy. Originally conceived as a way of increasing Black ownership of large companies, BEE (now called Broad‐Based BEE, or BBBEE) refers to a state policy whose objective is to racially transform the South African economy. Black business ownership remains one of the pillars of BBBEE (still commonly referred to as BEE), though now it encompasses not only large, but also mid‐sized and small businesses. The policy has also expanded to include other pillars such as management control, skills development, procurement, and the socio‐economic development of marginalized communities (Southall 2007a; Tangri and Southall 2008).
- *Free university education for all*: Like BEE, this is a policy that is targeted at the (emerging) middle class and elite. However, unlike BEE, it is framed in universal—that is, race‐neutral—terms.
- *Expanding unemployment insurance*: Like free university education, this is a policy that is framed in race‐neutral terms. Unlike free university education and BEE, it targets individuals lower on the stratification ladder.
- *Universal basic income:* We view this as an extreme case of universal social policies. Unlike the other policies we analyze, universal basic income is a policy that is not conditional on any individual characteristics, be they race, employment status, or university enrolment.

We presented respondents with each of these policies and asked them to evaluate whether they were (a) desirable and (b) viable. For each policy, we averaged respondents' perception of its desirability and viability, with 0 indicating the policy is neither viable nor desirable, 1 indicating the policy is both viable and desirable, and 0.5 indicating the policy is either desirable or viable but not both. We view this measure as stronger than an independent measure of desirability for both conceptual and methodological reasons. Conceptually, our goal is to capture policies that respondents would commit time and resources to implement. Policies viewed as desirable but not viable are less likely to meet this criterion than policies viewed as both viable and desirable (see López et al. 2022). Methodologically, we believe that combining elites' assessments of the desirability and viability of policies helps mitigate against social desirability biases that might be more pronounced in the former measure. Respondents who indicate that policies are desirable but not viable might do so for a range of different reasons, at least some of which might be related to their unwillingness to openly state that they find redistributive policies undesirable. That said, in the appendix (see Tables A1 and A2), we present findings separately for policy desirability and viability. Results are consistent with findings using the joint measure.

#### 4.3.2 Explanatory Variables

We have two main explanatory variables. The first captures respondents' *racialized identities*. Toward the end of our survey, we collected demographic data. We asked respondents how they would identify racially using official census categories: Black African, Coloured,^5^ Indian, White, or Other.^6^ Based on literature that points to a perception of “linked fate” between racialized minorities in different socio‐economic positions (Lacy 2007; Lu and Jones 2019), we expect elites to support policies that target individuals who share their racialized identities. More concretely, this means that we hypothesize that Black African, Coloured, and Indian elites will be more likely than White elites to support BEE.

Our second main explanatory variable measures respondents' *historical understandings of racialized inequality*. To capture the historicized view, we asked respondents to indicate their level of agreement with two statements on a five‐point scale, from completely disagree (1) to completely agree (5). The first statement read: *“White South Africans continue to benefit considerably from the advantages they got during apartheid.”* The second statement asked more broadly about racialized inequality, without mentioning the word “apartheid” but using the word “still” to imply a continuity between past and present: *“Inequality in South Africa is still very racialized.”*

We scaled both items into an index through a simple average to more reliably capture respondents' historical view of contemporary racialized inequality, even if both variables are de facto not correlated with each other (Pearson correlation of −0.07). We do so because we expect the items to capture different but complementary aspects of the broader concept of a historical view of racialized inequality. Based on literature indicating that individuals' redistribution preferences are related to their understandings of the past—and specifically, the relationship between past and present (Teeger 2014, 2024), we hypothesize that elites who advance a historical understanding of racialized inequality will be more likely to support race‐conscious redistributive policies than elites who do not advance this historical understanding.

We also include control variables for elite sector, survey year, party affiliation, sex, age, and education. We provide more detail on these variables in the appendix.

#### 4.3.3 Model Specification

Our OLS regression models are specified as follows:

$$Y_{i}=\alpha +\beta_{1}{\text{ELITE\_RACE}}_{i}+\beta_{2}\;{\text{HISTORICIZED\_VIEW}}_{i}+\beta^{T}\;X_{i}+\mu_{i}$$

Where Y_*i*_ represents our outcome variables, ELITE_RACE and HISTORICIZED_VIEW represent our main explanatory variables, X_*i*_ is a vector of control variables included to address potential confounding effects, *α* represents the intercept, *β* represents average effects, and *μ*_*i*_ represents the error term.

In the 2023 survey, we included questions designed to measure additional predictors of support for redistribution, such as fear of crime (Rueda and Stegmueller 2016), trust in institutions (Berens and Von Schiller 2017; Habibov et al. 2018), and views of meritocracy (Atria 2022; Kirsten and Biyase 2025). We ran a full model utilizing only the 2023 sample, including all controls, to test the robustness of our findings. Results still hold, and regression outputs can be found in the appendix, where we also present the distributions of all our variables (see Tables A3 and A4).

We conducted several other analyses to check the reliability and robustness of our findings. Our sample size is relatively large for an elite survey (López 2023a), given that elites are a small and difficult‐to‐reach population (Cousin et al. 2018; Hoffmann‐Lange 2018). Nonetheless, a survey with 326 observations and multiple covariates raises concerns about statistical power. Table A5 in the appendix presents results of a power analysis,^7^ which demonstrate that our models have high statistical power to detect effects of the magnitude observed. The appendix also includes results for models with our main outcome and explanatory variables, but without controls (see Table A6).

**FIGURE A1:**
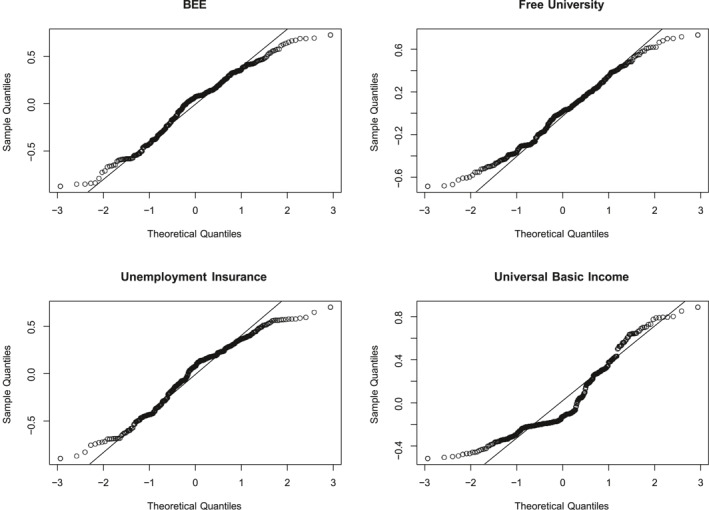
Normality of Residuals.

We also tested the regression models presented in Table 2 below for normality, heteroscedasticity, and multicollinearity. We utilize Shapiro‐Wilk test for the normality assumption (Royston 1982), the Breusch‐Pagan test for heteroscedasticity (Breusch and Pagan 1979), and the variance inflation test for multicollinearity (Fox and Monette 1992). We report results in the appendix (see Table A7). Residuals are approximately normal with some mild deviation in the ends (see Figure A1). Relatedly, the Breusch‐pagan test shows heteroscedasticity. We correct for this by estimating the OLS models with heteroscedasticity‐consistent standard errors.

**TABLE 2:** OLS models estimating support for race‐conscious and race‐neutral policies.

	Dependent variable:			
	BEE	Free university	Unemployment insurance	Universal basic income
	(1)	(2)	(3)	(4)
(race)BlackAfrican	0.204***	0.201***	0.138**	−0.083
	(0.071)	(0.068)	(0.067)	(0.061)
(race)Indian	0.125	0.068	0.187**	−0.085
	(0.092)	(0.079)	(0.086)	(0.066)
(race)Coloured	0.027	0.065	0.162*	−0.141
	(0.101)	(0.089)	(0.088)	(0.087)
Historicized view	0.317***	−0.040	−0.251**	0.033
	(0.118)	(0.113)	(0.127)	(0.111)
(year)2023	−0.137***	0.120**	0.205***	0.101**
	(0.050)	(0.051)	(0.057)	(0.049)
(sector)Business	0.134**	0.035	0.206***	0.014
	(0.061)	(0.055)	(0.059)	(0.049)
(sector)Political	0.038	0.0004	0.052	0.163**
	(0.074)	(0.069)	(0.075)	(0.069)
(party)ANC Membership	0.230***	0.053	0.213***	0.086
	(0.070)	(0.065)	(0.066)	(0.059)
(party)DA Membership	−0.134	−0.015	0.141**	−0.092
	(0.081)	(0.063)	(0.069)	(0.062)
(party)Other Memberships	0.007	−0.097	−0.101	0.015
	(0.092)	(0.073)	(0.090)	(0.079)
(sex)Male	−0.061	0.029	−0.056	−0.038
	(0.050)	(0.058)	(0.056)	(0.062)
Education	0.001	−0.043***	0.003	0.010
	(0.014)	(0.014)	(0.016)	(0.018)
Age	−0.001	−0.0002	0.001	0.002
	(0.003)	(0.002)	(0.003)	(0.002)
Constant	0.373*	0.692***	0.351	0.023
	(0.226)	(0.205)	(0.219)	(0.221)
Observations	303	301	303	302
*R* ^2^	0.294	0.177	0.214	0.115
Adjusted *R* ^2^	0.262	0.140	0.179	0.075
Residual Std. Error	0.367 (df = 289)	0.330 (df = 287)	0.368 (df = 289)	0.337 (df = 288)

*Note:* Robust standard errors in parentheses. Baseline: white, civil servants, female, no affiliation.

* *p <* 0.1.

** *p <* 0.05.

*** *p <* 0.01.

## 5 Findings

### 5.1 Racialized Identities and Historical Understandings of Racialized Inequality

**FIGURE 1:**
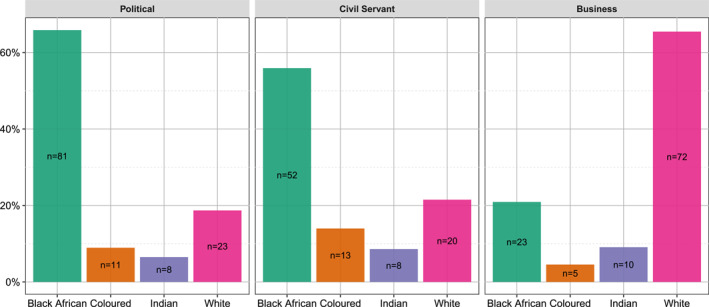
Elites' racialized identities, by sector (*n* = 326).

Figure 1 presents our sample by race and sector across both waves of our survey. Most respondents identified using one of the four Census categories, with only 10 choosing the “Other” option and two refusing to answer, suggesting that for elites, like for most South Africans, being asked to racially identify on a survey is an unremarkable exercise, with a straightforward answer. Although a product of colonization and apartheid, contemporary *racialized identities* in South Africa are, in other words, taken for granted. The distribution of our sample is consistent with what we know about elites in South Africa, with Black Africans (who make up 81.4% of the general population) comprising most of the political sector and Whites (who make up 7.3% of the general population) holding disproportionate power in the economic sector (Bond 2000; Reddy 2025; Thomas 2017).

**FIGURE 2:**
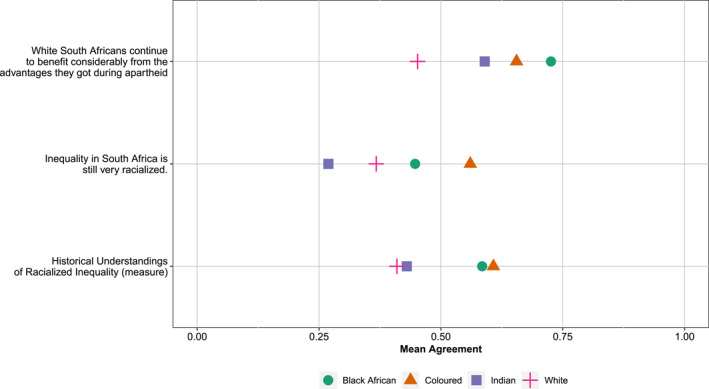
Historical understandings of racialized inequality, by elites' racialized identities. Items were normalized with 1 indicating the highest level of agreement.

While assessing the impact of individuals' own racialized identities on their policy preferences is an important component in understanding how the politics of redistribution is racialized, it is not the only one. People's assessment of the antecedents of racial disparities, specifically whether they believe that current disparities are the result of histories of racial oppression (see Bonilla‐Silva 2006; Teeger 2024), can also play an important role. Two items in our survey captured respondents' beliefs about the ongoing effects of South Africa's history of legislated racism on contemporary inequality, which we used to build a composite measure of *historical understandings of racialized inequality*, as described in Section 4.3. Figure 2 shows mean levels of agreement with the two statements as well as the composite measure.

Results show that, on average, Black African respondents advanced the historicized view more so than did White respondents, across all three measures. Indian respondents were, on average, closer to White respondents, and Coloured respondents were, on average, closer to Black African respondents. Coloured respondents were most likely to say that inequality is still very racialized in South Africa. Indian respondents were least likely to agree with this statement. However, when we asked a question focused explicitly on white privilege, using apartheid as an anchor, Black Africans were most likely to agree with this statement, and Whites were least likely to do so. Our small sample size of Indian and Coloured elites limits what we can say about these trends, but the trends do suggest that *how* we ask about the past matters for responses.

At the same time, the types of descriptive average effects that we present in Figure 2 obscure variation within racial groups. In the final empirical section of the paper, we leverage this variation to examine the relationship between elites' historical view of racialized inequality and their support for redistributive policies, controlling for their racialized identities. First, however, we discuss our outcome variables (support for policies framed in a. race‐neutral and b. race‐conscious terms) and present descriptive statistics of their association with our main explanatory variables (a. participants' racialized identities and b. their historical understanding of racialized inequality).

### 5.2 Racialized Policies

**FIGURE 3:**
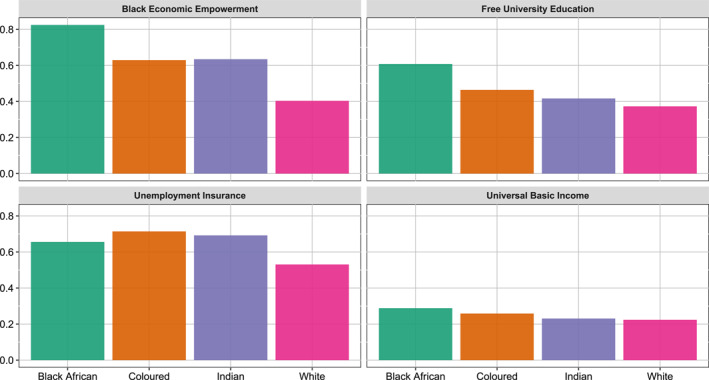
Support for race−conscious and race−neutral policies, by elites' racialized identities. Items were normalized with 1 indicating the highest level of agreement.

Racialized societies are, of course, not only about actors' racialized identities. They are configured out of racialized structures of inequality. Redistributive measures, too, can be racialized, with policies framed in race‐conscious or race‐neutral terms. Figure 3 presents respondents' average level of support, by race, for the four redistributive policies that constitute our outcome variables. One of these policies (BEE) is framed in explicitly race‐conscious terms. The other three (free university education, unemployment insurance, and universal basic income) are framed in race‐neutral terms.

Across the board, White elites show the lowest level of support for redistributive policies.^8^ Free university education for all—a race‐neutral policy targeting the (emerging) middle class and elite—receives less support overall than does BEE—a race‐conscious policy targeting a similar socio‐economic demographic. Unemployment insurance—a policy framed in race‐neutral terms that targets individuals lower on the socioeconomic distribution, is more popular than BEE among Coloured, Indian, and White elites. This is not the case for Black African elites who express greater levels of support for BEE. The basic income—the most universal of all the policies in that it does not specify any eligibility criteria, whether to do with race, education, or employment status—received the lowest level of support across the board.

**FIGURE 4:**
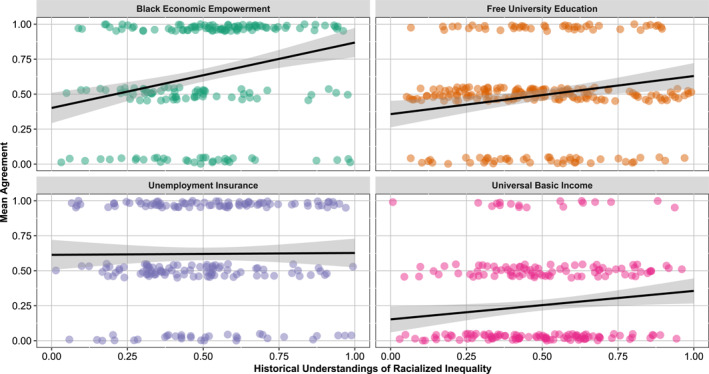
Historical understandings of racialized inequality by support for race−conscious and race−neutral policies. Items were normalized with 1 indicating the highest level of agreement. Observations were slightly jittered to improve visualization of overlapping cases. Jittering does not affect the correlation analysis.

When we look at the relationship between our second explanatory variable (historical understandings of racialized inequality) and policy support, we find a positive relationship between holding the historicized view and supporting redistributive policies, as shown in Figure 4.

All plots evidence an association between holding a historicized view and supporting redistributive policies, though the strength of the Pearson correlation is strongest for BEE (*R* = 0.27), followed by Free University Education (*R* = 0.18), Universal Basic Income (*R* = 0.13), and Unemployment Insurance (*R* = 0.037). This suggests that holding a historical view of racialized inequality increases support for redistribution, but more so for policies framed in race‐conscious terms.

Thus far, we have presented descriptive statistics, showing variation among our sample of South African elites by racialized identities; by endorsement of a historical view of racialized inequality; and by support for policies framed in race‐conscious and race‐neutral terms. In what follows, we present results from regression models that bring these variables together. We compare the effects of our explanatory variables on each of the policies that constitute our outcomes variables.

Table 2 presents four models that include the analytic categories we propose: racialized identities, historical understanding of racialized inequality, and racialized policies, as well as controls for elite sector, political affiliation, age, sex, education, and survey year. Model 1 shows the relationship between these variables and the race‐conscious policy of BEE. Models 2, 3, and 4 show the relationship between the same independent variables and three policies framed in race‐neutral terms.

Results show that elites' racialized identities structure their support for redistributive policies. Elites who identify as Black African are more supportive of the race‐conscious policy of BEE than are elites who identify as White. Our models fail to detect statistically significant differences between elites who identify as Coloured or Indian and those who identify as White. It is possible, therefore, that Coloured and Indian elites hold views that are indistinguishable from those held by White elites. If this is indeed the case, a potential explanation might have to do with discourses that suggest that these groups were not “White enough” during apartheid and are not “Black enough” in the democratic era (see Adhikari 2005)—in other words, Coloured and Indian respondents may feel that, although BEE is supposed to benefit all groups disadvantaged by apartheid, in practice, it does not benefit the racialized groups with which they identify. That said, we caution against overinterpreting these results due to the very small number of Coloured and Indian elites in our sample.

At the same time, Black African, Coloured, and Indian elites are all more supportive of expanding unemployment insurance than are White elites. Although framed in race‐neutral terms, beneficiaries of this policy are least likely to be White, given that they are least likely to be unemployed. Although not framed in explicitly race‐conscious terms, these findings nonetheless evidence support for a “linked fate” hypothesis, whereby elites are more likely to support policies whose beneficiaries share their racialized identities, as discussed above (see Lu and Jones 2019). A similar trend is not evidenced in the case of free university for all. Here, identifying as Black African is associated with positive support for this policy. However, we find no statistically significant relationship for respondents who identify as Coloured or Indian. We find a negative relationship between identifying as Black African, Coloured, or Indian on elites' support for the most universal of the policies (the universal basic income), although the effects do not reach statistical significance.

In addition to the effects of individuals' racialized identities, these models show that holding a historical view of racialized inequality has an effect on elites' redistributive preferences, as hypothesized based on the collective memory literature (see Teeger 2014, 2024). Specifically, elites who believe that South Africa's history continues to affect racialized inequality in the present are more likely to support the race‐conscious BEE policy than are elites who do not hold this historicized view, independent of the effect of their own racialized identities. Holding this view is negatively associated with support for unemployment insurance—a policy that, like BEE, is related to labor force participation, but is decidedly presentist.

In addition to the effects of our main explanatory variables, we find a statistically significant positive relationship between being a business elite and supporting unemployment insurance. We hypothesize that business elites are more willing to support unemployment insurance because they may perceive it as a way of outsourcing market risks to the state, while political and civil service elites may wish to avoid the complexity of this means‐tested policy and opt instead for other welfare policies. Similarly, there is a positive effect of being a member of the business elite on support for BEE, possibly because businesspeople have directly experienced this policy as viable in their own workplaces.

Importantly, even if expected, we find that party affiliation has explanatory power. Members of the ruling party, the ANC, are more likely to support BEE as compared with those without political affiliation. Being a member of the DA has a negative effect on support for this policy, as compared with those who have no political affiliation—though the difference fails to reach statistical significance. In contrast, members of both the ANC and the DA show support for unemployment insurance, indicating perhaps that this is a policy around which there is a degree of cross‐party agreement. There is a small, but statistically significant, negative relationship between education and support for free university for all. This perhaps reflects a view by those who attended university when it was not free that it is possible to do so (an “if we did it, you can do it” type of argument). Finally, our year variable captures the extent to which overall support for different policies changed between 2014 and 2023. We find that in 2023, there's less support for BEE policies, but support for all other policies increased on average. It's possible that this has to do with several corruption scandals around BEE between these two points in time (see Buthelezi and Vale 2023; Southall 2007b).

## 6 Discussion and Conclusions

Reddy (2025: 287) has recently argued that “[t]he extent to which race patterns the political opinions of the elite class is fundamentally an empirical question. Currently, it's largely unanswered. But it seems a given that should the research ever be done, its impact will be shown to be sizeable, however measured.” Focusing on South Africa—a country with high levels of racial inequality but an increasingly racially diverse elite—we take on this research agenda by outlining three ways that researchers can center race in their analysis of elites' redistributive preferences. First, and most obviously, researchers can look at the role that elites' own *racialized identities* might play in their views on inequality. Second, they can attend to elites' *historical view of racialized inequality* by asking whether or not elites view current inequality as structured by the legacies of state‐sanctioned racism. Finally, they can attend to the *racialization of policies* by examining elites' willingness to support policies framed in explicitly race‐conscious terms versus those framed in race‐neutral terms.

We exemplified this approach by drawing on an original data set collected from surveys of South African political, civil service, and economic elites. Our findings show that elites' own racialized identities matter for their willingness to support redistributive policies. As important as their racialized identities, however, is elites' historical perspective on racialized inequality. Elites who viewed current inequality as the result of the country's racist past were much more likely to support explicitly race‐conscious policies than were elites who did not hold such a historical view. This was true regardless of elites' own racialized identities.

That racialized minorities would support race‐conscious redistribution is not self‐evident, nor should we think that it is a finding that holds across time and place. Elites are often invested in narratives of individualism, meritocracy, and even their own ordinariness, eschewing discussions of privilege and disadvantage (Khan 2011; Reeves and Friedman 2024; Rivera 2016). Such narratives legitimize their positions of power and help pass on privilege from one generation to the next. At the same time, research shows that racialized minorities are often acutely aware of discrimination that follows them into the most powerful of positions (J. L. Hochschild 1996; Pittman Claytor 2020), resulting in more collective frames of “linked fate” that stress the persistence of racism across the stratification system. Other evidence, however, shows that this is not always the case and that racial and ethnic minorities can become strong proponents of ideologies of meritocracy as they take up positions of power (Saini et al. 2023).

Indeed, our study finds a complex relationship between elites' racialized identities and their support for redistributive policies. Black Africans, Coloureds and Indians were more supportive than Whites of unemployment insurance—a policy framed in race‐neutral terms, but whose beneficiaries are more likely to be racialized minorities. Black Africans were also more supportive of the explicitly race‐conscious policy of BEE than were Whites, but the full model, with controls, revealed no statistically significant relationship between identifying as Coloured or Indian and supporting BEE. As noted, due to small sample sizes for these two groups, we caution against overinterpreting these results.

It is also not self‐evident that individuals who hold a historical view of racialized inequality would automatically support race‐conscious policies to redress these inequities. Indeed, scholars have argued that the best way to address racial inequalities is to advance race‐neutral policies that would receive broad support (see Wade 2024; Wade and Moreno‐Figueroa 2021; Wilson, 2000). A rising tide, as the saying goes, lifts all boats. Our data suggest that elites in South Africa did not hold this view, in spite of the fact that many of the race‐neutral policies we presented them with would, in practice, primarily benefit those disadvantaged by apartheid. Instead, the historicized view served as a strong predictor of elites' support for the explicitly race‐conscious policy of BEE.

Although our findings may not generalize to other cases in a statistical sense, we view our study as offering important avenues for other researchers concerned with how race matters for elites' support for redistribution. In taking forward a research agenda on race and elites' redistributive preferences, we encourage researchers to attend not only to elites' own racialized identities, but also to broader processes of racialization that might structure elites' support for redistributive processes. As we have demonstrated, such processes include the framing of policies (in race‐neutral or race‐conscious terms) and the construction of historical understandings of contemporary racialized inequality (which draw on—or eschew—racially oppressive pasts as causal factors structuring contemporary inequality).

Recent moves in the United States and elsewhere to purge histories of racial oppression from national memory (see Da Silva and Larkins 2019; Smith 2025) highlight how collective memory is not just about the past. Instead, such attacks are about delegitimizing redress policies in the present. As race scholars have argued, racial inequality is sustained through color‐blind discourses that deny the ongoing effects of histories of racial oppression. When individuals posit an equal playing field where none exists, they thwart policies aimed at redressing historical injustices and provide ideological support for maintaining an unequal status quo (see Bonilla‐Silva 2006; Ray 2023; Teeger 2024). Our paper adds to this conversation by demonstrating how elites' historical undestandings of racialized inequality shape their redistributive policy preferences. In doing so, it highlights the importance of attending to when and how elites learn about histories of racial oppression and their connection to the present.

One source of historical knowledge is personal and familial experience of legislated racism. Elites of color in South Africa undoubtedly have access to this source, as they inhabit a world that is temporally close to the country's apartheid past. However, our findings show that elites' historical understandings of racialized inequality have an independent effect on their policy preferences, which is not fully captured by their racialized identities. Future research should examine the role of institutions—such as schools and universities—in fostering elites' historical consciousness. Existing evidence points to the power of color‐blind discourses in such spaces (Ansell 2004; Teeger 2024). However, the variation in elites' historical views and policy positions indicates that further research is needed to unpack why some South African elites see continuities between past and present, while others eschew such connections. Such studies will be especially pressing as time passes and elites' autobiographical access to the era of legislated apartheid racism recedes. Here, we encourage future researchers to attend to whether South African elites' racialized identities continue to structure their policy preferences as well as to whether these effects are mediated by elites' historical understanding of racialized inequality.

The South African case reminds us that elite formation is not a static process but can change over time. By centering race in our analysis, we can begin to untangle when and how increased diversity in elite composition might lead to different redistributive preferences and, ultimately, policies.

## Ethics Statement

The study received approval from the LSE Research Ethics Committee (REC ref. 39573).

## Conflicts of Interest

The authors declare no conflicts of interest.

## Data Availability

Research data are not shared due to the small sample population and risks of particular configurations of demographic variables being uniquely identifying.

## Endnotes

^1^
For a definition of redistributive policies that centres the role of the state, see Alesina and Ferrara (2005).

^2^
This is not to say that elite composition is static in cases where there has not been a political transition to democracy. For example, as Reeves and Friedman (2024) show, even the composition of the British elite—often assumed to be extremely rigid and hereditary—has shifted over time, albeit it alongside enduring processes of elite reproduction.

^3^
For recent work that combines positional, income‐based, and reputational approaches to elite studies, see Reeves and Friedman (2024).

^4^
At the time of our surveys, the ANC was the ruling party and the DA was the official opposition party. In the most recent national elections (2024), the ANC's vote share dropped below 50% for the first time since democracy to 40.18%. The DA was broadly stable at 21.81% (up 1.04% from the previous election). Following this election, the ANC and DA, along with several smaller parties, entered into a Government of National Unity.

^5^
On the meaning of Coloured identity and the historical construction of this distinct racial category in South Africa, see Dooms and Chutel (2023) and Erasmus (2001).

^6^
The census uses apartheid‐era racial categories, which remain salient in how South Africans continue to identify in the democratic era (see Posel 2001).

^7^
We used the R package pwr (Champely et al. 2020), which implements Power Analysis as defined by Cohen (2013).

^8^
In the appendix, we present t‐tests of differences in mean support for each of the policies between Whites and Black Africans (Table A8), Whites and Coloureds (Table A9), and Whites and Indians (Table A10). Results show that differences between these groups are statistically significant for BEE at the 0.05 level. Results vary for other policies. As shown in Table 2, once we run the full models, with controls, we no longer detect statistically significant results for Coloured or Indian elites' support for BEE. Given the small number of respondents in these sub‐samples, we caution against overinterpreting results for these two groups. Differences in policy support between Black Africans and Whites are robust across models.

## Appendix

**TABLE A1:** Logistic models for policy desirability.

	Dependent variable			
	BEE	Free university	Unemployment insurance	Universal basic income
	(1)	(2)	(3)	(4)
(race)Black African	1.315***	1.232***	1.030**	0.212
	(0.412)	(0.426)	(0.418)	(0.419)
(race)Indian	0.634	0.239	0.904	−0.309
	(0.554)	(0.560)	(0.594)	(0.504)
(race)Coloured	0.023	0.086	1.120*	−0.995
	(0.560)	(0.559)	(0.647)	(0.612)
Historicized view	1.230*	−0.896	−0.918	−0.103
	(0.728)	(0.818)	(0.826)	(0.747)
(year)2023	−0.400	1.157***	0.599	1.038***
	(0.375)	(0.389)	(0.393)	(0.331)
(sector)Business	0.809**	0.156	0.738*	0.144
	(0.382)	(0.400)	(0.392)	(0.382)
(sector)Political	−0.304	−0.517	−0.403	1.043**
	(0.595)	(0.519)	(0.616)	(0.506)
(party)ANC Membership	1.887***	0.380	1.585***	0.184
	(0.632)	(0.484)	(0.587)	(0.469)
(party)DA Membership	−0.097	−0.090	1.102**	−0.469
	(0.494)	(0.495)	(0.539)	(0.475)
(party)Other Memberships	−0.094	−1.345**	−0.775	−0.628
	(0.553)	(0.537)	(0.543)	(0.645)
(sex)Male	−0.385	0.244	−0.023	0.005
	(0.403)	(0.429)	(0.392)	(0.345)
Education	0.034	−0.288**	0.129	0.135
	(0.117)	(0.124)	(0.107)	(0.103)
Age	0.016	−0.009	0.018	0.009
	(0.019)	(0.017)	(0.016)	(0.016)
Constant	−1.617	3.407**	−2.140	−3.059**
	(1.530)	(1.634)	(1.439)	(1.437)
Observations	304	306	305	303
Log Likelihood	−148.718	−158.584	−154.955	−177.958
Akaike Inf. Crit.	325.435	345.167	337.911	383.916

*Note:* Robust standard errors (HC1) in parentheses. Baseline: white for race, civil servants for sector, female for sex, and no affiliation for party.

* *p* < 0.1.

** *p* < 0.05.

*** *p* < 0.01.

**TABLE A2:** Logistic models for policy viability.

	Dependent variable			
	BEE	Free University	Unemployment Insurance	Universal Basic Income
	(1)	(2)	(3)	(4)
(race)Black African	0.743*	1.164**	0.325	−2.182***
	(0.401)	(0.544)	(0.426)	(0.650)
(race)Indian	0.451	0.855	0.981*	−1.220
	(0.515)	(0.627)	(0.585)	(0.869)
(race)Coloured	−0.162	0.594	0.547	−1.145
	(0.528)	(0.692)	(0.602)	(0.971)
Historicized View	2.466***	0.275	−1.710**	0.656
	(0.835)	(0.869)	(0.775)	(0.966)
(year)2023	−1.222***	0.317	1.504***	−0.189
	(0.371)	(0.375)	(0.352)	(0.451)
(sector)Business	0.687*	0.207	1.369***	0.345
	(0.364)	(0.418)	(0.374)	(0.546)
(sector)Politicals	0.611	0.368	0.599	1.178
	(0.523)	(0.530)	(0.471)	(0.731)
(party)ANC Membership	1.135**	0.184	1.050**	1.524*
	(0.481)	(0.490)	(0.439)	(0.822)
(party)DA Membership	−1.080**	−0.045	0.491	−0.408
	(0.533)	(0.542)	(0.462)	(0.689)
(party)Other Memberships	0.276	0.398	−0.357	1.514**
	(0.565)	(0.551)	(0.578)	(0.718)
(sex)Male	−0.448	−0.017	−0.528	−0.571
	(0.377)	(0.388)	(0.359)	(0.503)
Education	−0.030	−0.206**	−0.063	−0.041
	(0.107)	(0.103)	(0.095)	(0.133)
Age	−0.019	0.008	−0.012	0.005
	(0.018)	(0.017)	(0.016)	(0.019)
Constant	0.148	−1.032	0.340	−1.807
	(1.537)	(1.482)	(1.391)	(1.860)
Observations	303	302	304	304
Log Likelihood	−161.825	−154.399	−171.401	−109.344
Akaike Inf. Crit.	351.650	336.797	370.802	246.689

*Note:* Robust standard errors (HC1) in parentheses. Baseline categories: white (race), civil servants (sector), female (sex), no affiliation (party).

∗ *p <* 0.1.

∗∗ *p <* 0.05.

∗∗∗ *p <* 0.01.

**TABLE A3:** OLS models with additional controls (available for 2023 only).

	Dependent variable			
	BEE	Free university	Unemployment insurance	Universal basic income
	(1)	(2)	(3)	(4)
(race)Black African	0.215**	0.293**	−0.021	−0.210*
	(0.105)	(0.119)	(0.128)	(0.119)
(race)Indian	0.109	0.073	0.038	−0.170
	(0.117)	(0.137)	(0.134)	(0.125)
(race)Coloured	0.058	0.184	0.090	−0.321**
	(0.116)	(0.130)	(0.128)	(0.143)
Historicized view	0.430***	−0.014	−0.191	0.055
	(0.137)	(0.173)	(0.180)	(0.156)
(sector)Business	0.326***	0.118	0.247**	0.128
	(0.121)	(0.113)	(0.121)	(0.114)
(sector)Political	0.107	−0.009	0.015	0.314**
	(0.142)	(0.128)	(0.138)	(0.138)
(party)ANC Membership	0.170	−0.067	0.209	−0.147
	(0.133)	(0.124)	(0.128)	(0.131)
(party)DA Membership	−0.205*	−0.084	0.017	−0.350***
	(0.119)	(0.093)	(0.101)	(0.130)
(party)Other Memberships	0.057	−0.004	−0.207	−0.114
	(0.095)	(0.098)	(0.128)	(0.127)
(sex)Male	−0.109*	0.011	0.027	0.006
	(0.056)	(0.065)	(0.066)	(0.078)
Education	−0.014	−0.020	−0.045*	0.014
	(0.020)	(0.021)	(0.025)	(0.024)
Age	0.007**	0.003	−0.003	−0.002
	(0.003)	(0.004)	(0.004)	(0.004)
Fear of crime	−0.047	0.129	0.310*	0.142
	(0.178)	(0.176)	(0.175)	(0.179)
Believe in meritocracy	−0.024	0.217	0.101	0.098
	(0.113)	(0.139)	(0.164)	(0.172)
Trust in institutions	0.346**	0.252	0.481**	0.347*
	(0.150)	(0.182)	(0.203)	(0.189)
Constant	−0.280	0.031	0.619	0.012
	(0.397)	(0.345)	(0.391)	(0.414)
Observations	132	129	133	132
*R* ^2^	0.600	0.231	0.373	0.139
Adjusted *R* ^2^	0.548	0.129	0.292	0.027
Residual Std. Error	0.288 (df = 116)	0.315 (df = 113)	0.334 (df = 117)	0.360 (df = 116)

*Note:* Robust standard errors (HC1) in parentheses. Baseline categories are white for race; civil servants for sector; female for sex; and no affiliation for party.

* *p* < 0.1.

** *p* < 0.05.

*** *p* < 0.01.

**TABLE A4:** Distribution of key variables.

Variable	Median	SD	Min	Max	Missing
Black Economic Empowerment	0.50	0.42	0	1	5
Free university	0.50	0.36	0	1	7
Unemployment insurance	0.50	0.40	0	1	4
Universal basic income	0.50	0.35	0	1	6
Historical understandings of inequality	0.50	0.24	0	1	2
Age	52.00	9.40	24	80	12
Education	9.00	1.55	1	11	6
Elite merit	0.77	0.25	0	1	2
Fear of crime	0.88	0.20	0	1	1
Trust in institutions	0.58	0.21	0	1	1

*Note:* Elite Merit, Fear of Crime, and Trust in Institutions are only available for the 2023 sample.

**TABLE A5:** Power analysis for OLS models with 14 predictors.

Model	N	R2	f2	Power
BEE	303	0.294	0.416	1.000
Free university	301	0.177	0.215	1.000
Unemployment insurance	303	0.214	0.272	1.000
Universal basic income	302	0.115	0.130	0.993

**TABLE A6:** OLS Models Predicting Support for Race Conscious or Race Neutral Redistribution (w/o controls).

	Dependent variable			
	BEE	Free university	Unemployment insurance	Universal basic income
	(1)	(2)	(3)	(4)
(race)BlackAfrican	0.380***	0.210***	0.145***	0.035
	(0.050)	(0.045)	(0.053)	(0.046)
(race)Indian	0.212**	0.041	0.151*	−0.027
	(0.084)	(0.077)	(0.089)	(0.077)
(race)Coloured	0.179**	0.067	0.202**	−0.0005
	(0.084)	(0.074)	(0.087)	(0.075)
Historicized view	0.224**	0.127	−0.091	0.178*
	(0.098)	(0.090)	(0.103)	(0.091)
Constant	0.312***	0.321***	0.568***	0.151***
	(0.053)	(0.049)	(0.057)	(0.050)
Observations	319	318	320	318
*R* ^2^	0.213	0.097	0.031	0.021
Adjusted *R* ^2^	0.203	0.086	0.018	0.009
Residual Std. Error	0.380 (df = 314)	0.343 (df = 313)	0.402 (df = 315)	0.351 (df = 313)

*Note:* Baseline category is white for race.

* *p* < 0.1.

** *p* < 0.05.

*** *p* < 0.01.

**TABLE A7:** OLS assumption checks: Normality, heteroscedasticity, and multicollinearity.

Model	Shapiro‐Wilk p	Breusch‐Pagan p	Mean VIF
BEE	0.0001	0.0000	1.58
Free university	0.0047	0.0579	1.57
Unemployment insurance	0.0000	0.0182	1.57
Universal basic income	0.0000	0.0034	1.58

**TABLE A8:** *T*‐Tests Comparing Policy Support Index (White vs. Black African Respondents).

	Policy	Mean_White	Mean_Black	t_statistic	*p*_value
1	BEE	0.404	0.825	*−*8.826	0
2	Free university	0.373	0.608	*−*5.635	0
3	Unemployment insurance	0.531	0.656	*−*2.443	0.015
4	Universal basic income	0.224	0.288	*−*1.458	0.146

**TABLE A9:** *T*‐Tests Comparing Policy Support Index (White vs. Coloured Respondents).

	Policy	Mean_White	Mean_Coloured	t_statistic	*p*_value
1	BEE	0.404	0.630	*−*2.366	0.023
2	Free university	0.373	0.464	*−*1.165	0.252
3	Unemployment insurance	0.531	0.714	−2.155	0.037
4	Universal basic income	0.224	0.259	−0.434	0*.666*

**TABLE A10:** *T*‐Tests Comparing Policy Support Index (White vs. Indian Respondents).

	Policy	Mean_White	Mean_Indian	t_statistic	*p*_value
1	BEE	0.404	0.635	*−*2.557	0.015
2	Free university	0.373	0.417	*−*0.611	0.545
3	Unemployment insurance	0.531	0.692	*−*1.915	0.062
4	Universal basic income	0.224	0.231	*−*0.098	0.922

Description of Control Variables included in main models.

- *Elite sector:* captures whether respondents are in the business, political, or civil service sector with dummy variables using civil servants as the reference category.
- *Survey year*: a dummy variable with 2014 as the reference category to account for changes over time in support for redistribution.
- *Party Affiliation:* political party affiliation is likely correlated with elites' ideological positions on issues of race and redistribution. We control for party affiliation with dummy variables where “no party affiliation” is the reference category.
- *Sex:* a dummy variable with 0 = female and 1 = male.
- *Age*: respondents' age could imply different political socialization and memories of apartheid. We use respondents' year of birth to control for these potential effects.
- *Education*: Respondents' educational background may broadly affect their views on redistribution, but it may also affect their views on particular policies, such as the provision of free university education. We utilize an ordinal scale ranging from no formal schooling (1) to PhD (11) to control for respondents' level of education.

Description of Control Variables Only Available in 2023.

- *Elite Merit:* respondents were asked to rate the main cause of their accomplishments with (1) indicating it was all due to personal effort and (10) indicating it was all due to opportunities others did not have. We normalized the variable to range from 0 to 1
- *Fear of Crime:* respondents were asked how concerned they were about crime, with (1) indicating respondents were not at all concerned and (10) indicating they were very concerned. We normalized the variable to range from 0 to 1.
- *Trust in Institutions:* respondents were asked how much they trust (a) the courts; (b) parliament, and (c) the national executive, on a scale ranging from (1) do not trust at all (10) fully trust. We averaged the result of all three items and normalized them to create a variable that captures respondents' level of trust in institutions, ranging from 0 to 1.
